# Risk Stratified Follow-Up for Endometrial Cancer: The Clinicians’ Perspective

**DOI:** 10.3390/curroncol30020173

**Published:** 2023-02-13

**Authors:** Anumithra Amirthanayagam, Louise Boulter, Nessa Millet, Hilary J. McDermott, Jo Morrison, Alexandra Taylor, Tracie Miles, Lorna Coton, Esther L. Moss

**Affiliations:** 1 Leicester Cancer Research Centre, University of Leicester, Leicester LE1 7RH, UK; 2 Department of Gynaecological Oncology, University Hospitals of Leicester NHS Trust, Leicester LE1 5WW, UK; 3 School of Sport, Exercise and Health Sciences, Loughborough University, Loughborough LE11 3TU, UK; 4 Somerset NHS Foundation Trust, Taunton TA1 5DA, UK; 5 The Royal Marsden NHS Foundation Trust, London SW3 6JJ, UK; 6 Royal United Hospital Bath NHS Foundation Trust, Bath BA1 3NG, UK

**Keywords:** endometrial cancer, follow-up, survivorship, patient-initiated follow-up, telephone follow-up

## Abstract

Risk-stratified follow-up for endometrial cancer (EC) is being introduced in many cancer centres; however, there appears to be diversity in the structure and availability of schemes across the UK. This study aimed to investigate clinicians’ and clinical specialist nurses’ (CNS) experiences of follow-up schemes for EC, including patient-initiated follow-up (PIFU), telephone follow-up (TFU) and clinician-led hospital follow-up (HFU). A mixed-methods study was conducted, consisting of an online questionnaire to CNSs, an audience survey of participants attending a national “Personalising Endometrial Cancer Follow-up” educational meeting, and qualitative semi-structured telephone interviews with clinicians involved in the follow-up of EC. Thematic analysis identified three main themes to describe clinicians’ views: appropriate patient selection; changing from HFU to PIFU schemes; and the future of EC follow-up schemes. Many participants reported that the COVID-19 pandemic impacted EC follow-up by accelerating the transition to PIFU/TFU. Overall, there was increasing support for non-HFU schemes for patients who have completed primary treatment of EC; however, barriers were identified for non-English-speaking patients and those who had communication challenges. Given the good long-term outcome associated with EC, greater focus is needed to develop resources to support patients post-treatment and individualise follow-up according to patients’ personal needs and preferences.

## 1. Introduction

Endometrial cancer (EC) is the most common gynaecological cancer in the UK, with more than 9700 new cases per year [1]. Almost half (48%) of EC cases are categorised into the low- or intermediate-risk groups [2], which are typically associated with excellent long-term survival, 10-year overall survival (94.1% and 84.5%, respectively) [3], thus leading to an ever-increasing prevalence of EC survivors.

The prognostic ability of EC classification has led to the development of innovative follow-up strategies for cases where cancer recurrence is a rare event. In particular, patient-initiated (PIFU) and telephone follow-up (TFU) have the aim of reducing routine hospital follow-up (HFU) appointments and encouraging symptom self-management for patients [4], in keeping with the Living with and Beyond Cancer and National Survivorship policies [5]. PIFU and TFU have been reported to be acceptable to patients [6,7], and PIFU is associated with significant cost savings for both the patient and the healthcare economy [8,9]. There are negative aspects associated with such schemes, in particular the patients’ ability and/or willingness to contact the specialist team if symptoms develop. Additionally, an increased fear of cancer recurrence and increased General Practitioner (GP) contacts have been reported with PIFU as compared to HFU [10]. The British Gynaecological Cancer Society (BGCS) [11] is supportive of innovative follow-up schemes for EC, and risk-stratified follow-up has become a core component of Personalised Stratified Follow Up (PSFU) pathways [12]; however, the uptake and structure of schemes varies across the UK [13]. The geographical distribution, innovative follow-up scheme design and uptake are dependent upon the local clinical teams; however, in the majority of cases, Clinical Nurse Specialists (CNS) lead the management of the schemes, since they are most commonly the primary point of contact for patients.

Intensive follow-up for EC has not been shown to impact overall EC survival [14], and the COVID-19 pandemic has reportedly accelerated the uptake of virtual follow-up appointments in order to avoid hospital visits for asymptomatic patients with no suspicion of recurrence. As a result [15], many health care professionals now have gained experience of innovative follow-up schemes to manage patients following EC treatment.

The aim of this study was to identify the current views and experiences of clinicians on innovative follow-up schemes for EC, the challenges and future direction of such schemes, including biomarker monitoring, and the adoption of a UK-wide risk-stratified follow-up scheme.

## 2. Materials and Methods

A sequential explanatory mixed methods study was conducted consisting of: (1) online questionnaire to CNSs; (2) audience survey of participants attending a national “Personalising Endometrial Cancer Follow-up” meeting; and (3) qualitative semi-structured telephone interviews with clinicians involved in the follow-up of EC. Ethical approval was granted by the University of Leicester Research Ethics Committee (27210 and 28874). All participants consented to participate in the study: Part 1 consent was at the start of the questionnaire; Part 2 written consent was taken as part of the meeting registration; and, for Part 3, written consent was taken and an interview scheduled at a date/time convenient for the participant.

Online questionnaire: The online questionnaire was designed to explore CNS’s experiences and views on innovative follow-up schemes (Supplementary Table S1). In particular, questions asked their views on the structure of schemes, practical challenges encountered when managing patients and the training that they had received to undertake their role. In addition to rating scale questions, free-text boxes were included to enable participants to elaborate on their responses. The questionnaire was piloted through the BGCS nurses’ group and distributed through the BGCS nurses’ group membership. A reminder email was sent after 3 weeks, and the link to the survey remained open for 5 weeks.

Audience survey: A virtual study day focusing on “Personalising Endometrial Cancer Follow-up” was hosted by the University of Leicester in October 2020. A series of presentations were given by national experts, including “Current evidence for EC follow-up schemes”, “Management of recurrent EC” and “Role of survivorship programmes”. Meeting registration was free and advertised to clinicians through the BGCS. Prior to the meeting, the poll questions were verified as appropriate and piloted amongst the meeting speakers and specialists with an interest in EC follow-up, to ensure clarity and remove potential bias. An online audience participation survey was conducted in real time using the Slido app (Cisco Systems) during the meeting. The survey asked questions on preferred follow-up scheme design, the optimum time to start a patient on a scheme and patient selection (Supplementary Table S2).

**Figure 1:**
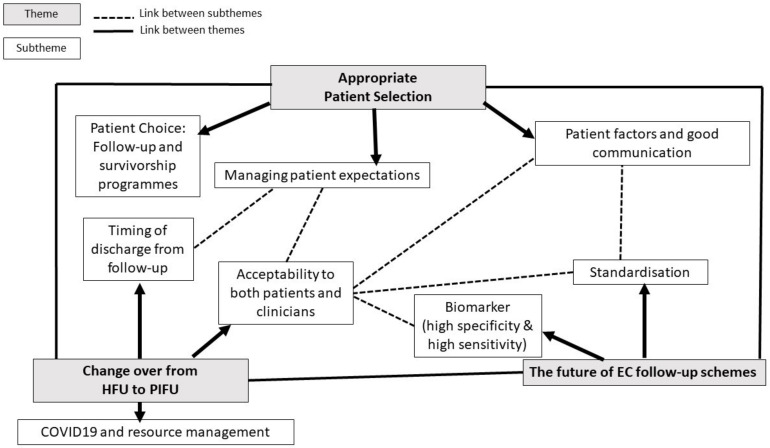
Themes and subthemes identified from the semi-structured interviews (*n* = 29).

Qualitative interviews: Semi-structured interviews were conducted within a high specificity target sample of gynaecology clinical nurse specialists (CNSs), consultant subspecialist gynaecological oncologists (GOs), cancer unit lead gynaecologists (ULGs) and medical/clinical oncologists (ONCs), between May and September 2021. Questions focused on addressing/identifying the main variations in practice across the UK and hurdles to changing practices; participants were asked to reflect on the pros and cons experienced by those that pioneered innovative follow-up schemes prior to the formal recommendation as guidelines. Questions were included on the role of PIFU as a follow-up scheme and the potential impact of a biomarker to detect EC recurrence on the future of EC follow-up. A qualitative semi-structured interview guide was informed by the medical literature on EC follow-up, the responses to the online questionnaire/survey responses and guideline recommendations. The questions were piloted to ensure understanding of questions with specialists with an interest in EC follow-up (Supplementary Figure S1). Stratified and purposeful sampling was applied to ensure that the views gathered spanned diverse clinical backgrounds, experience and geography throughout the UK. The interviews were conducted virtually by telephone by one researcher (AA), a senior gynaecology trainee with an interest in gynaecological oncology who had undergone training in conducting/analysing qualitative research. The interviews were digitally recorded using an encrypted audio recorder and transcribed verbatim. Field notes were taken to identify points to challenge the participant to ensure the empirical data obtained had maximum information power. Regular appraisal of the empirical data and quality of information power dictated the need for further recruitment and modifications to sampling criteria/specificity of the sample. Data collection was deemed complete when no adjustments needed to be made to the sample size to accommodate new themes/knowledge to enhance information power. All transcripts were read through twice, with the aim of identifying themes using a deductive approach, and coded by the lead researcher (AA) with a subset of interview data coded by a second researcher (ELM) to enhance rigor and trustworthiness of the data analysis. Discussions were held to acknowledge the differences in codes and resolve and incorporate the nuances in interpretation. The codes were organised into a thematic tree with three main themes and associated sub-themes supported by participant quotes (Figure 1).

## 3. Results

### 3.1. Online Questionnaire

Responses were received from 22 CNSs, of whom half worked only in a cancer centre (Supplementary Table S1). Twenty of the respondents reported that their cancer centre/unit had an innovative follow-up scheme in place, with nurse-led TFUbeing the most common scheme, followed by nurse-led clinical follow-up and PIFU. Seventy percent of the participants reported that, in their opinion, the COVID-19 pandemic had had an impact on their clinic service by causing accelerated transition to PIFUor TFU. The majority of CNSs had been in their post for less than 10 years (72.7%), and 17 of the 22 respondents reported not having undertaken specific training to support them in managing patients on follow-up schemes. Additional training in the management of late radiation effects and psychosexual issues were highlighted as the main areas of interest for additional training.

### 3.2. Audience Survey

Participants included CNSs (47%), GOs (16%), ULGs (7%). Twenty-seven percent of the audience identified as “other”, including oncologists, research nurses, academics and gynaecology nurses. PIFU was the most popular alternative follow-up scheme (52%), and the majority view was that an alternative follow-up scheme for low-risk EC should be standard of care (66%) (Supplementary Table S2). There was strong support for a structured survivorship programme following completion of EC treatments, with 89% of the opinion that attendance at a programme was beneficial for patients. Similarly, the participants were very supportive of the development of a standardised, national follow-up scheme for EC (97%). It was felt that biomarker monitoring would increase clinician confidence in transferring patients to an alternative scheme (90% agreed), but such a marker would need to have high sensitivity (90–95% level) to detect recurrence.

### 3.3. Qualitative Interviews

In total, 33 clinicians responded to the study invitation; of these, 29 participated in an interview: 10 GOs, six ULGs, six ONCs and seven CNSs. The median interview duration was 20 min, ranging from 12 to 46 min. Thematic analysis identified three main themes to explain the views on EC follow-up: appropriate patient selection; changing from HFU to PIFU schemes; and the future of EC follow-up schemes (Figure 1).

#### 3.3.1. Theme 1: Appropriate Patient Selection

##### Subtheme: Managing Patient Expectations

Patient anxiety and expectations often influence patient satisfaction outcomes. It was felt that higher rates of satisfaction were necessary for successful implementation of a PIFU scheme, i.e., less resistance to change. Many participants reported that their departments preferred to offer a range of follow-up options, thereby enabling patients to decide their preferred option and allowing transfer between schemes at any point of their care: “If it is appropriate, I will offer it to them, I don’t make decisions on their behalf” (GO9). The need to remind patients about the scheme and symptoms that should prompt contact was raised by several participants, especially in units where the follow-up scheme was a new development, and concern was expressed that cancer recurrences may not be detected in a timely manner. Conversely, the possibility that PIFU could prompt patients to contact the specialist team as soon as they had symptoms rather than waiting until a scheduled HFU appointment to report symptoms was mentioned by one participant: “I’ve had maybe two patients who actually had recurrence; they had bleeding, but they did not contact us but waited until their appointment” (GO8). This highlighted the importance of involving patients in their management, since the success of PIFU is highly dependent on patients buying into the concept, since the onus of seeking medical advice is placed on them. It also allows them to express any concerns they might have as to why a PIFU scheme may not be suitable for them.

Particularly during the transition period (HFUto TFU/PIFU), a more open approach was preferred by patients and clinicians. This was reinforced by participants who worked in centres/units who had pioneered the use of such schemes prior to the published BGCS guidance, as they had already passed this transition phase and successfully implemented PIFU.

##### Subtheme: Patient Characteristics and Good Communication

The loss of patients to follow-up, language barriers and concerns that patients may not seek help appropriately were raised as important considerations when selecting patients for PIFU. Certain patient factors, particularly patients whose primary language was not English or had comorbidities, such as dementia or mental health conditions, that could result in communication difficulties, were seen as being at high risk of being disadvantaged, by not being able to report symptoms to their specialist team:

*“If they don’t speak English fluently, then they can’t be on patient-initiated follow-up, because they need to understand obviously if something goes wrong, or [if] they have a worry they need to be able to contact us to discuss their concerns, and if they can’t speak English then there is no way that we can contact them.” (CNS 9)*

*“Elderly patients who can’t really assess themselves and too anxious patients, patients who have got psychiatric illness—these are the two types of patients we can’t really analyse—because anxiety is something where they are anxious for each and everything you know and if we don’t give them follow-up, they just climb up the wall.” (GO2)*

In addition, it was felt that some patients just did not want to engage with self-management and concerns were raised that they would not contact the specialist team if symptoms developed:

*“We do have some ladies who don’t want to be a bother and if you don’t ask, they won’t tell you until they are really struggling.” (GO8)*

*“You need to look after the psychological aspect—if you just leave them on PIFU—if they suffer in silence, you wouldn’t know.” (GO2)*

The identification of such patient factors and attitudes was highlighted as important for maintaining patient safety, and that a “one size fits all” approach is not feasible and that no one scheme would be suitable for the whole EC survivor population.

##### Subtheme: Survivor’s Choice: Follow-Up and Survivorship Programmes

There was universal support for patients to attend a survivorship course following completion of primary treatment that covered the common post-treatment/diagnosis challenges experienced by patients. Many participants reported that a survivorship programme was not available at their institution, with a lack of resources or staff as the primary reasons:

*“They certainly should have ‘survivorship programme’ for every type of cancer. As cancer treatment gets better, you are creating a huge cohort of patients who are surviving cancer, living with the aftermath of the cancer … complications and side effects … they cannot revert back to where they were.” (GO7)*

Concerns were raised as to whether offering patients with low-risk EC participation in a survivorship programme might inhibit their ability to move forward from this episode in their life after successfully completing treatment, given the typical good long-term prognosis. However, other participants raised the issue of the need to manage post-treatment effects:

*“… when the treatment is done and the cancer is treated, then they are living with the aftermath of what has been done and quite a few times I have seen people who have actually struggled to cope with surgeries, complications, side effects from surgery.” (GO3)*

Hence, patient selection was identified as a key requirement of having a successful and effective cancer survivorship programme.

#### 3.3.2. Theme 2: Change from HFU to PIFU

##### Subtheme: Acceptance to Both Patients and Clinicians

There was overwhelmingly positive support for moving patients away from regular clinician-led HFU. The majority of respondents reported that innovative follow-up schemes had already been introduced in their hospital, although the format of the schemes adopted varied between TFU or a hybrid TFU/PIFU scheme, rather than PIFU alone. In some cases, participants used the term PIFU to refer generically to innovate follow-up schemes; therefore, whenever this term was used, its meaning was clarified by the interviewer.

The reported advantages of transferring patients to such schemes included enabling clinician time and resources to be focused on patients with red-flag symptoms where a clinical examination was indicated. Furthermore, there was also the view that PIFU could encourage patients to return to normality sooner. The anxiety experienced by some patients, when attending even routine HFU appointments, was also mentioned and was seen as a positive of TFU/PIFU:

*“Most of these patients would be doing quite well but when they get the letter about their appointment or when they have to come, that’s when a lot of patients say that ‘I don’t remember I had this disease’ and—when they come for the interview or assessment—’that’s when I get stressed’.” (GO7)*

It was felt that PIFU was particularly suitable for low-risk EC, in keeping with the results from the audience survey, since it was felt that it could encourage patients to put their diagnosis/treatment behind them by removing the schedule of hospital appointments that had a very low likelihood of detecting cancer recurrence: “They do not have to worry about taking leave … or attending clinic or looking for parking” (CNS9). The open access format of PIFU was felt to keep the lines of communication open with the patient should they have a concern. In addition, the financial losses and/or work absences incurred by patients and their family members to enable them to attend a hospital appointment were also felt to be a consideration and were frequently cited as a positive aspect of PIFU.

The majority of GO participants expressed the view that an asymptomatic pelvic recurrence identified on clinical examination was very rare; therefore, PIFU was acceptable and cost-effective: “You need to examine 700 patients to detect one recurrence that is asymptomatic” (GO5). However, several ONC participants expressed the view that face-to-face appointments in the early stages post-surgical and -medical treatment were valuable for recovery; however, they did highlight that oncologists were likely to see patients within the higher-risk categories as compared to GO/UGL participants.

##### Subtheme: Appropriate Time for Discharge/Transfer to PIFU

The main variation noted was the timing of discharge to primary care and/or transfer to PIFU. The most frequently reported duration of follow-up was 5 years; however, some departments had earlier discharge for low-risk EC at 3 years, and other participants reported that their departments discharged patients to primary care after one face-to-face appointment post-surgery.

After completion of 5 years of follow-up and discharge to primary care, a number of clinicians still recommended that the gynaecological oncology services were the first point of contact should the patient experience red-flag symptoms rather than a suspected cancer referral appointment through their GP:

*“I advise patients to contact our CNS team rather than go through GP, as I feel it would be better managed than waiting for the referral process.” (ULG6)*

These personal preferences and variations to follow-up schemes suggest that there is an element of clinician bias/comfort level, i.e., patient acceptance is not the only factor and clinician acceptance is also important. The concern raised was that a local EC recurrence in patients discharged to primary care may take time to be referred back to the gynaecological oncology team, which could impact the treatment options available and the patient’s long-term outcome. Again, there were conflicting opinions between ONC and GO participants on the optimum timing of discharge, which could be attributed to differences in the patient characteristics and risk categorisation seen by the two groups of clinicians.

##### Subtheme: COVID 19 Impact on the NHS Resource Management

The COVID-19 pandemic was identified as having a dramatic impact on many aspects of healthcare, including reducing hospital appointments for patients who are classified as clinically vulnerable. It was cited as a major factor in aiding the transition to PIFU, and support for non-face-to-face follow-up was reported as being well received by both patients and hospital management: “I do a mixed approach with face-to-face and telephone, especially with COVID, and I think patients appreciate this” (ONC1). It was reported that elderly patients and those who had undergone adjuvant treatment were very keen to reduce any activities that could increase their risk of exposure to a COVID-19 infection: ‘’Really positive impact—we were going to pilot nurse-led telephone FU but COVID rocketed us forward. I have had great feedback from patients” (online survey, CNS) and “It has meant that patients are more amenable to telephone/patient-initiated as they are not physically coming to clinic due to the pandemic” (online survey, CNS). Clinicians also reported being reassured knowing that virtual clinics were being introduced in all aspects of healthcare, and that COVID-19 had reduced the pressure of having to justify why the change is needed.

#### 3.3.3. Theme 3: The Future of EC Follow-Up Schemes

##### Subtheme: Standardisation

A standardised approach to follow-up was discussed. Half of the participants were of the opinion that the recent BGCS guidelines were sufficient to enable standardisation across the UK, since they contained options for personalising care. Concerns were expressed that the introduction of more structured follow-up protocols could lead to patient dissatisfaction, and the communication between clinicians and patients could feel one-sided with no room for flexibility:

*“The disadvantage is if it is applied as a blanket policy, we should have the discussion with the patients and take into account their concerns, other risk factors, which might prevent them from presenting to you; this is how it should be for every patient.” (GO7)*

The other half of participants were in favour of a national, standardised approach to follow-up, since it was felt that this approach could reduce patient anxiety by removing discrepancies in models of care between hospitals and regions, as well as support clinicians’ decisions from a medico-legal perspective:

*“The main advantage is that it should give the patients confidence and the clinicians confidence that this is what we should do; there would be recurrences regardless of how early-stage or low-grade they are and it’s easier if we have a national protocol.” (GO7)*

CNSs in particular were strongly in favour of a structured follow-up protocol, and clinicians acknowledged that, as CNSs were the first point of contact for PIFU schemes, clear guidance would help support them and streamline the workload.

##### Subtheme: A Biomarker to Detect EC Recurrence

The future role of a biomarker with a high sensitivity/specificity to detect EC recurrence was discussed. The majority of the clinicians were supportive of the theoretical use of such a biomarker and the potential of its use in a remote monitoring scheme for EC follow-up. However, a number of participants were concerned that regular blood tests might increase patient anxiety, particularly in low-risk EC. Other participants felt that a highly specific biomarker would instil greater patient and clinician confidence in moving onto a PIFU or remote monitoring scheme. Several participants were apprehensive as to the potential impact on patient management in the absence of efficacious or salvage treatments for EC recurrence.

*“If you are detecting a recurrence early, then you need to demonstrate that your intervention is acceptable—it’s going to improve quality and quantity of life.” (GO10)*

*“We have to be realistic, so for some of those patients there is not much value to detect an early recurrence because the therapeutic options are not there, so you have to be selective—so which ones do we want to monitor?” (GO1)*

ONCs were the group most supportive of such a development, with the view that the benefits of potential curative management outweighed the inconvenience of regular testing and patient anxiety being reported. The need for the biomarker to undergo extensive real-world testing before implementation was discussed:

*“Is there a way to detect it early i.e., at a stage where it is salvageable—is the marker capable of doing that? We want good sensitivity and good specificity. Sensitivity would give more confidence.” (ONC1)*

*“If you have a test that has high specificity rather than sensitivity—because you will end up screening half the people, if you have high sensitivity—I think that would be useful.” (GO9)*

Whilst the sample of clinicians interviewed have expressed keen interest in the potential benefits of a biomarker, the key concerns regarding treatment options and increasing patient anxiety remained.

## 4. Discussion

In this study, we aimed to capture the views of a wide range of clinicians and CNSs involved in the follow-up of patients after EC treatment, particularly exploring experiences of innovative follow-up schemes. The results give real-world insights into the barriers and advantages to such schemes, which can be used to optimise current schemes but can also inform future developments in EC follow-up, particularly the introduction of survivorship schemes and biomarker monitoring.

The most common theme identified was appropriate patient selection and, although PIFU was identified as the preferred follow-up scheme structure, many participants reported numerous barriers that prevented patient uptake. TFU was seen as an intermediate step for both patients and clinicians, increasing confidence in a move away from HFU. The reported experience from the UK is that the majority of EC recurrences are associated with symptoms [16]—for example, vaginal vault recurrences (25 of 29 cases) and distant recurrences (20 of 20 cases) [17]. In particular, the number of asymptomatic recurrences in low-risk EC is reported to be very low—for example, 0.33%—with the majority being local recurrences (10 of 12 cases) [18], which is associated with high salvage rates, 5-year OS 68% (95% CI 59–75%) [19]. These retrospective reports have supported the introduction of innovative follow-up schemes that do not include regular clinical examination, and the ability of patients to identify and report symptoms was highlighted as a major consideration for recruitment to PIFU. This concern has been also raised by healthcare professionals on the introduction of PIFU schemes in head and neck cancer [20,21]. In particular, language was commonly cited as a barrier to PIFU/TFU participation due to concerns that patients would not be able to self-report symptoms. Multi-lingual clinical staff within the specialist team can overcome this as a barrier, as reported in the Leicester PIFU scheme, where one CNS was a Guajarati speaker [6]. To overcome language barriers, alternative methods of communication, such as mobile apps that contain commonly used phrases translated into different languages, have been shown to improve patient-clinician communication [22]. Since Asian ethnicity are the largest ethnic minority group diagnosed with uterine cancer in the UK (4.1% [23]), and given the increase in cases being diagnosed in women of Asian/Asian British ethnicity [2], consideration should be given to the development of resources that can support non-English-speaking populations to participate in PIFU schemes.

Opinions on the timing and structure of innovative follow-up schemes differed between subspecialties, with oncologists more supportive of the need for regular follow-up post-treatment rather than an immediate transfer to PIFU. The differences in opinion could be as a result of the different risk profile of cases seen by the two groups, and reinforces the need for follow-up schedules to be personalised to the patient’s risk of recurrence. The BGCS guidelines [11] do support a range of options for PIFU, depending on the risk classification, thereby enabling clinicians to determine the option that they feel is most appropriate for their patients. The importance of clinician autonomy was highlighted in views on the development of a more standardised UK follow-up scheme. Opinions on the duration of follow-up varied widely and, although many HFU schemes have been 5-years in duration, given that the majority of recurrences occur within the first 2 years of diagnosis, many participants were supportive of shorter duration schemes. The time to recurrence has been shown to differ with risk classification, with the peak time to recurrence for low-risk EC being reported as year 4–6 [3]. Therefore, shorter-duration schemes may result in the majority of low-risk EC recurrences being diagnosed after a patient has been discharged from specialist follow-up. This possibility had been considered by many participants, and the need for education of both EC survivors and GPs as to the possibility of late recurrences was raised in order to expedite investigations and assessment by the gynaecological oncology team. A study of healthcare professionals by Williamson et al. reported that a greater role for primary care was viewed as a positive development for cancer follow-up, although the need for close working between primary and secondary care, particularly with access to specialist advice, was highlighted [24]. Given the developments in treatments available for recurrent EC [25], including immunotherapy [26] and secondary cytoreductive surgery [27], this is likely to become more of a focus in the future, and further research is needed to investigate whether the time from symptomatic recurrence to treatment has an impact on the overall survival. The role of a biomarker that could predict EC recurrence, such as circulating tumour DNA [28], was of interest to clinicians, but the need for high sensitivity/specificity and for available treatment options was highlighted.

Post-treatment educational courses, commonly termed survivorship courses, are reported to be beneficial in supporting patients’ return to everyday life. Course content often covers topics such as fatigue management, fear of cancer recurrence (FCR) and body image, and attendance has been shown to reduce anxiety in women following EC treatment [29]. Survivorship courses appear to be viewed more positively than Health Needs Assessments, reported to cause confusion [30], and Survivorship Care Plans, which are reported to have a negative impact on quality of life, anxiety and depression [31]. The challenges already discussed regarding accessibility by ethnic minority or marginalised groups also apply to survivorship schemes, and work is needed to develop resources that are accessible to non-English-speaking populations. Many of the available survivorship courses are not tumour-specific and, as a result, the life-changing symptoms reported following EC treatment [32], including urinary/faecal incontinence, lymphoedema (leg swelling) and pelvic pain, are often not covered in detail. This can leave patients attending such courses with unmet information needs and a lack of self-management strategies to address symptoms. Specific symptoms, such as FCR, may benefit from the input of a psycho-oncologist, although access to such specialist services in not universal, and the development of cognitive behavioural therapy interventions may be of an alternative option for some patients [33]. Given the rising EC incidence, good long-term survival and geographical disparities of available courses, consideration should be given to the development of an EC-specific survivorship course, focusing on the specific needs of this patient cohort.

The COVID-19 pandemic has had far-reaching impacts on all aspects of healthcare, including reportedly playing a significant role in accelerating the uptake of PIFU, with the instigation of new schemes, but also in the increased acceptability by patients and clinicians. In a patient survey of experiences with a newly established TFU scheme in response to the COVID-19 situation, patient satisfaction was high, 83%, rating as excellent [34]. The results of the study also highlighted patient views on clinical examination as an investigation for EC follow-up, since 76% reported that not having an examination did not affect their appointment, suggesting that it is the communication aspect of a consultation that is viewed as being of greater value to patients. Patient examination as part of EC follow-up is known to cause great anxiety in some patients [35]; therefore, the development of schemes which do not include regular clinical examination may lead to greater patient satisfaction.

A point made by several participants was the ability of the clinical staff to adapt resources in order to support patients during the COVID-19 pandemic restrictions. The need for appropriate training for CNSs to support them in the management of innovative follow-up schemes appears to be lacking, and many CNSs reported interest in attending targeted educational events. This lack of a formal training programme has been reported in other studies [36], and may have been the rationale for greater support by the CNSs interviewed for a structured, national survivorship scheme. Given the wide variation in experience, diverse skills and needs required to undertake a CNS role, a more formal educational programme may be of interest, covering the key aspects of EC survivorship to help CNSs to feel more supported in their role.

### Limitations

This study aimed to explore the experiences of clinicians and CNSs involved in everyday care of EC survivors. The three different methods were used in order to maximise the number of participants; however, as with any qualitative or survey study, the opinions expressed cannot be taken as representative of the gynaecological oncology community as a whole. A few of the participants from cohort 1 and 2 may have also participated in an interview. Many of the challenges experienced in the structure of the healthcare system may be UK-focused, rather than being internationally applicable, and the study population did not include GPs, so we were not able to explore their views of PIFU and the challenges experienced in primary care.

## 5. Conclusions

There is increasing clinician support for non-HFU schemes for patients who have completed primary treatment of EC. There are barriers to the uptake of PIFU by patients who have communication or language challenges, and there is a lack of technological developments to facilitate patient-clinician communication. Despite strong support for a survivorship course, many clinicians report that such schemes were not available for their patients. Given the good long-term outcome associated with EC, greater focus is needed to develop resources to support patients post-treatment and individualize follow-up depending on patients’ personal needs.

## Data Availability

The data are not available due to ethical restrictions.

## Supplementary Materials

The following supporting information can be downloaded at: https://www.mdpi.com/article/10.3390/curroncol30020173/s1, Table S1: Online Questionnaire to Gynaecological oncology Clinical Nurse; Table S2: Results of Audience Survey; Figure S1: Interview Schedule.
