# Supplementary material for: Risk Stratified Follow-Up for Endometrial Cancer: The Clinicians’ Perspective
Source: Curr Oncol. 2023 Feb 13;30(2):2237–48. doi: 10.3390/curroncol30020173 (PMC9955652; doi:10.3390/curroncol30020173)
Supplement: Supplementary file 1 [file curroncol-30-00173-s001.zip › curroncol-2171800-supplementary.pdf]

Current Oncology

Article

Risk Stratified Follow-Up for Endometrial Cancer: The Clinicians' Perspective

Anumithra Amirthanayagam [1], Louise Boulter [2], Nessa Millet [1], Hilary J. McDermott [3], Jo Morrison [4], Alexandra Taylor [5], Tracie Miles [6], Lorna Coton [6] and Esther L. Moss [1,2,*]

Table S1. Online Questionnaire to Gynaecological oncology Clinical Nurse Specialists (n=22).

Question	Results
Are you a Clinical Nurse Specialist in gynaecology?	Yes 96% (22/23)<br>No (4%) (1/23)
How long have you been in your current post?	Median = 5 months<br>Minimum = 12 months<br>Maximum = 370 months
How many Clinical Nurse Specialists (including yourself) work in your team?	Median = 2 CNS<br>Minimum = 1 CNS<br>Maximum = 4 CNS
Where do you work?	Cancer centre 10 (45.5%)<br>Cancer unit 8 (36.4%)<br>Cancer Centre and unit 3 (13.6%)
Does your centre/unit follow any guidelines for the follow-up of endometrial cancer?	BGCS 15/22 (68.2%)<br>Regional Cancer Network 14/22 (63.6%)<br>ESGO 5/22 (22.7%)<br>Local Cancer Centre/Unit 8/22 (36.4%)<br>*BGCS only 5/22(22.7%)*<br>*Remainder used a combination of above options 17/22 (77.3%)*
If you have local guidelines for your cancer centre/unit, how often are these updated?	Annually 5/22 (22.7%)<br>Unsure/not known 3/22 (13.6%)<br>Less frequently than 1 year 4/22 (18.2%)<br>Unanswered 10/22 (45.4%)
Who is responsible for updating the local guidelines that your centre/unit uses	Both lead clinician and CNS 9/22 (40.9%)<br>Both lead clinician, CNS and different members of the team 2/22 (9.1%)<br>Clinical Lead (medical) 6/22 (27.3%)<br>Different members of the team 1/22 (4.5%)<br>Other (Network / Gynaecological Oncology Cancer Alliance Group) 2/22 (9.1%)
Who provides survivorship support for patients who have undergone treatment for endometrial cancer at your cancer centre/unit?	CNS only 6/22 (27.3%)<br>Macmillan Support worker only 1/22 (4.5%)<br>CNS & Macmillan Support worker 6/22 (27.3%)<br>CNS & Gynaecologist (unit/centre) 2/22 (9.1%)<br>CNS, Gynaecologist (unit & centre), Oncologist (medical/clinical) 1/22 (4.5%)<br>CNS, Macmillan Support worker, Gynaecologist (unit & centre), Oncologist (medical/clinical) 4/22 (18.2%)

Curr. Oncol. **2023**, *30*, 2237–2249. https://doi.org/10.3390/curroncol30020173 www.mdpi.com/journal/curroncol

Does your cancer centre/unit offer a structured survivorship course for patients following completion of treatment for endometrial cancer?	Yes (cancer centre) 7/22 (31.8%)<br>Yes (cancer unit) 3/22 (13.6%)<br>No (cancer centre) 4/22 (18.2%)<br>No (cancer unit) 6/22 (27.3%)<br>Other (local cancer support centre / Gynaecology wellbeing days) 2/22 (9.1%)
If yes, who runs the survivorship course	CNS only 3/10 (30%)<br>CNS and Macmillan Support worker 3/10 (30%)<br>Macmillan Support worker only 2/10 (20%)<br>CNS & Other (surgeon) 1/10 (1%)<br>Cancer Charity 1/10 (1%)
Does your cancer centre/unit offer reduced face-to-face clinician follow up for patients diagnosed with endometrial cancer?	Yes – early stage endometrial cancer 2/22 (9.1%)<br>Yes – early stage, low grade endometrial cancer 9/22 (40.9%)<br>Yes- stage/grade dependent on patients' needs and wishes 1/22 (4.5%)<br>Yes- all stages/grades of endometrial cancer 4/22 (18.2%)<br>Yes (early stage, low grade endometrial cancer & stage/grade dependent on patients' needs and wishes) 3/22 (13.6%)<br>No 2/22 (9.1%)<br>Other 1/22 (4.5%) PIFU
What type of follow-up model does your centre/unit use?	Nurse- led clinical follow-up only 2/22 (9.1%)<br>Patient initiated follow-up 5/22 (22.7%)<br>Nurse led telephone follow-up 3/22 (13.6%)<br>Nurse led telephone & clinical follow-up 3/22 (13.6%)<br>Doctor led telephone follow-up 2/22 (9.1%)<br>Nurse led telephone follow-up & early discharge to primary care 1/22 (4.5%)<br>Nurse-led clinical follow-up and Patient initiated follow-up 1/22 (4.5%)<br>Nurse-led clinical/telephone follow-up and Patient initiated follow-up 2/22 (9.1%)<br>Not Applicable 1/22 (4.5%)
If early discharge to primary care, how soon after completion of treatment are patients discharged?	6 weeks (only once response to Early discharge to primary care)
How long has this scheme been running at your centre/unit? Have any other schemes been used previously?	*15 responses collected*<br>Less than 6 months 1/15 (6.7%)<br>Between 6-12 months 2/15 (13.3%)<br>>=2 years 5/15 (33.3%)<br>>=5 years 6/15 (40%)<br>Unknown 1/15 (6.7%)
Do you keep a database of all patients added to an alternative follow-up scheme?	Yes – paper records and electronic database – generic 6/22 (27.2%)<br>Yes – electronic database – generic 3/22 (13.6%)<br>Yes – electronic database – specific for follow-up 7/22 (31.8%)

	Yes – electronic database – generic & specific for follow-up 1/22 (4.5%)<br>No- 2/22 (9.1%)<br>Not applicable & unanswered 2/22 (9.1%)<br>Other (own record) 1/22 (4.5%)
Do you keep documentation of contacts from patients on alternative follow-up schemes?	Yes – paper records and electronic database – generic 2/22 (9.1%)<br>Yes paper records & Other (appointment records) 1/22 (4.5%<br>Yes – electronic database – generic 6/22 (27.2%)<br>Yes – electronic database – specific for follow-up 4/22 (18.2%)<br>Yes – electronic database – generic & specific for follow-up 1/22 (4.5%)<br>No- 1/22 (4.5%)<br>Not applicable 2/22 (9.1%)<br>Other 1/22 (4.5%)
Do the Clinical Nurse Specialists have administrative support to help with running the scheme?	Yes 6/22 (27.2%)<br>No 12/22 (54.5%)<br>Not applicable or Unanswered 4/22 (18.2%)
Are there any aspects of the alternative follow-up scheme at your centre/unit that you would like to change?	Free text comments
Are there any aspects that you have found patients particularly struggle with if transferred to an alternative follow-up scheme?	Free text comments
In your opinion is there an alternative follow-up model that patients prefer or dislike more than others? Please give details	Free text comments
Do you have peer support from another clinical nurse specialist in managing the scheme? If not where do you go for support?	Free text comments
Do you think the COVID-19 situation has changed your attitude to the follow-up of endometrial cancer? If so how?	Free text comments
Has the COVID-19 situation changed the way that you and your centre/unit for asymptomatic endometrial cancer patients (no suspicion of recurrence) that typically attend for hospital follow-up appointments?	Changed follow-up appointment to telephone follow-up (CNS) 5/22 (22.7%)<br>Changed follow-up appointment to telephone follow-up (Doctors) 6/22 (27.2%)<br>Changed follow-up appointment to telephone follow-up (CNS & Doctors) 4/22 (18.2%)<br>Changed follow-up appointment to Patient initiated follow-up 2/22 (9.1%)<br>Stopped face-to face follow-up appointments 1/22 (4.5%)

	Stopped face-to face follow-up appointments & Changed follow-up appointment to telephone follow-up & Patient initiated follow up 1/22 (4.5%)<br>Stopped face-to face follow-up appointments & Changed follow-up appointment to telephone follow-up (CNS/Doctors) 1/22 (4.5%)<br>Other 1/22 (4.5%)
Have you noticed a change in the contact from patients on follow-up for endometrial cancer during the COVID-19 situation?	Yes, More patient contact/greater patient anxiety due to covid<br>No, patients trying to avoid attending hospital<br>No
Have you undergone any specific training to manage patients on the alternative follow-up scheme in place at your cancer centre/unit?	(free text – comments very varied answers not enough to mark as a percentage
Are there any aspects of the follow-up scheme and endometrial cancer survivorship that you do not feel you are trained to manage?	Free text comments
Are there any aspects of patient follow-up and survivorship in endometrial cancer that you would like to have additional training on?	Free text comments
Are there any aspects of patient follow-up and survivorship in endometrial cancer that you would like to have additional training on?	Free text comments
How would you prefer any additional training to be delivered?	
Would you be interested in undertaking a post-graduate qualification in cancer survivorship?	Yes 5/22<br>Yes (distance learning only) 6/22<br>Maybe 8/22<br>Other 3/22

Table S2. Results of Audience Survey (n=83)*.

Question	Response (%)
Which patients should be offered reduced face-to-face clinician follow up following primary treatment for endometrial cancer?	All stages & grades (10%)<br>Stage 1, Low Risk only (56%)<br>Stage I, all risk (6%)<br>Selected patients of any stage or risk (25%)<br>None (2%)<br>Don't know (2%)
Which is the best alternative follow-up scheme instead of face-to-face clinician follow-up following primary treatment for endometrial cancer?	Patient Initiated Follow-up (52%)<br>Nurse- led telephone Follow-up (29%)<br>Doctor-led telephone Follow-up (2%)<br>Nurse-led clinical Follow-up (12%)<br>Follow-up in primary care (2%)<br>Reduced schedule clinician Follow-up (3%)<br>Don't Know (0%)

How soon after completing treatment for low-risk endometrial cancer should patients be transferred to an alternative follow-up scheme	3months (50%)<br>Immediately OR within 6 months (18% each)<br>1 year (13%)<br>2 years (2%)<br>3 years/ Don't Know / Never (0%)
How soon after completing treatment for high-risk endometrial cancer should patients be transferred to an alternative follow-up scheme?	Two years (54%)<br>Three years (16%)<br>Never (11%)<br>Immediately (3%)<br>1 year (10%)<br>3 months (5%)<br>6 months (0%)<br>Don't know (0%)
Is attendance at a structured survivorship programme beneficial to patients after completing treatment for endometrial cancer?	Yes (89%)<br>No (5%)<br>Don't know (7%)
Should regular imaging (CT/MRI) be included in an alternative FU scheme for low risk endometrial cancer?	Yes (19%)<br>No (73%)<br>Don't know (8%)
Should regular imaging (CT/MRI) be included in an alternative FU scheme for high-risk endometrial cancer?	Yes (71%)<br>No (21%)<br>Don't know (8%)
Should an alternative FU scheme for low risk endometrial cancer be standard of care or optional?	Yes (66%)<br>Optional (34%)<br>Not offered (0%)
Should alternative FU scheme for high risk endometrial cancer be standard of care?	Optional (59%)<br>Standard of care (40%)<br>Not offered (2%)
Would you be more confident in transferring patients to an alternative FU scheme if they were able to have monitoring with a tumour marker that could detect recurrence?	Yes (90%)<br>No (5%)<br>Don't know (5%)
What detection level would be needed for a tumour marker?	100 % (2%)<br>95% (45%)<br>90% (29%)<br>85% (13%)<br>75% (5%)<br>50% (6%)
Would the development of a standardised, national alternative follow up scheme be of interest to your cancer centre/unit?	Yes (97%)<br>No (2%)<br>Don't know (2%)
Would you be interested in participating in a clinical trial of a remote monitoring scheme using a tumour marker versus clinician-led follow-up?	Yes – All stages and grades (44%)<br>Yes (Stage 1 low risk only) (23%)<br>Yes (Stage 1 all-risk only) (5%)<br>Yes selected patients at any stage or risk (11%)<br>No (4%)<br>Don't know (14%)

* not all participants answered every question

Figure S1. Interview Schedule.

1) What is your current role? Prompt: oncologist; gynaecological oncologist; gynaecologist (unit lead); CNS; other
2) Where do you work? Prompt: Cancer centre; cancer unit; both; other
3) Are you actively involved in the follow-up care of EC patients? Prompt: can you describe how? Joint clinic?
4) Is there a stratified risk follow-up for EC at your hospital at present? If yes, what model is it? Prompt: Telephone follow-up; patient-initiated follow-up; early discharge to GP; other. * if No go to question 8
5) What are the criteria for patients to be placed on this follow-up pathway? Prompt: Low risk only; early stage only; all stages/risk; selected patients; other
6) What do you see as the benefits of such a scheme? Do you think this view is shared by the colleagues in your team?
7) Have you found any problems with such a scheme? Do you think this view is shared by the colleagues in your team?
8) What are your thoughts on the different risk-stratified follow-up models eg telephone or patient-initiated follow-up? In your view do they have any particular benefits or problems?
9) Which is your preferred model? What are the reasons for your choice?
10) When in your view is the optimum time to discharge patients back to primary care after completion of treatment for endometrial cancer? Prompt: After treatment; 3 months; 1 years; 5 years; never
11) How do you see the future of endometrial cancer follow? Prompt: will risk stratified follow-schemes be expanded?
12) Do you see a potential role for a national standardised risk stratified endometrial cancer follow-up scheme with a standard protocol and management pathways? What in your view are the advantages or disadvantages of such a scheme?
13) Do you feel that a survivorship programme designed specifically for endometrial cancer would be something that would be of interest to patients? Is it something that you would be happy to recommend to patients?
14) If a tumour marker with a high sensitivity to detect endometrial cancer recurrence was available, could you see a role for a remote monitoring scheme, similar to the use of PSA in prostate cancer monitoring? Would it increase your confidence in transferring patients from routine clinical follow-up? What in your view would be the advantages and disadvantages of remote monitoring?
15) Should a tumour marker be available, how do you see the structure of a remote monitoring scheme? Would it benefit from having a way of patients contacting their clinical nurse specialist?

16) Do you think there will be any barriers to acceptance of a remote monitoring scheme by patients? By clinicians?

17) Are there any other points you would like to make?
